# Case Report: Intravascular Large B-Cell Lymphoma: A Clinicopathologic Study of Four Cases With Review of Additional 331 Cases in the Literature

**DOI:** 10.3389/fonc.2022.883141

**Published:** 2022-05-13

**Authors:** Yingying Han, Qingjiao Li, Dan Wang, Lushan Peng, Tao Huang, Chunlin Ou, Keda Yang, Junpu Wang

**Affiliations:** ^1^Department of Pathology, Xiangya Hospital, Central South University, Changsha, China; ^2^Department of Pathology, School of Basic Medicine, Central South University, Changsha, China; ^3^National Clinical Research Center for Geriatric Disorders, Xiangya Hospital, Central South University, Changsha, China; ^4^Department of Pathology, Hunan Provincial People’s Hospital, The First Affiliated Hospital of Hunan Normal University, Changsha, China

**Keywords:** Intravascular large B-cell lymphoma (IVLBCL), clinicopathologic feature, hemopathy, 331 cases, case report

## Abstract

Intravascular large B-cell lymphoma (IVLBCL) is a rare and highly malignant non-Hodgkin B-cell lymphoma with uncommon clinical presentation and poor prognosis. The diagnostic pitfall of IVLBCL is mainly due to the fact that subtle histological changes could be easily overlooked, in addition to its rare occurrence, non-specific and variable clinical presentations, and the absence of significant mass lesions. The purpose of this study is to further explore the clinicopathologic and molecular features of IVLBCL to ensure an accurate diagnosis of this entity. Here, we retrospectively present the data of the four new cases and the literature cases. The age ranged from 23 to 92, with a medium age of 67 and a male-to-female ratio of 1:1. The clinical manifestations are extremely variable, including fever, night sweats, weight loss, anemia, thrombocytopenia, unexplained hypoxemia, impaired consciousness, and skin lesions, as well as the extremely low levels of serum albumin, high levels of serum lactate dehydrogenase (LDH), soluble interleukin-2 receptor (sIL2R), and ferritin. Morphologically, 99.9% of cases showed a selective growth pattern with large, atypical lymphocytes within the lumen of small blood vessels. In addition, vast majority of cases were positive for CD20, CD79a, PAX5, MUM1, and BCL6, and a subset of cases expressed BCL2 and CD5, whereas CD3 and CD10 were typically negative. Ki-67 proliferative index ranged from 20% to 100%. To sum up, we have conducted comprehensive case reports, to the best of our knowledge, this is the largest reported cohort of IVLBCL cases. Comprehensive assessments and more IVLBCL cases are required for early diagnosis and prompt treatment.

## Introduction

Intravascular large B-cell lymphoma (IVLBCL) is a rare and distinct subtype of extranidal diffuse large B-cell lymphoma (DLBCL) and was first described in 1959 (1), and since then, more than 300 cases have been reported in the literature, mostly case reports and small series. It is characterized by tumor cells accumulation in the lumens of small and medium vessels, but there are rare cases characterized by proliferation in large blood vessels (2). Clinical presentations of IVLBCL are extremely various, including unexplained fever, night sweat, anemia, weight loss, impaired consciousness, and skin lesions. Diagnostic awareness deficiency and variable clinical behavior of IVLBCL potentially lead to delayed treatment and poor prognosis. In this study, we retrospectively analyzed the comprehensive clinical, laboratory, imaging, morphologic, immunophenotypic, and molecular characteristics of IVLBCL using data on four cases diagnosed at our hospital and additional 311 cases from the literature, to further improve the full recognition of the clinicopathologic characteristics of IVLBCL to ensure accurate diagnosis of this entity and facilitate appropriate treatment.

## Materials and Methods

### Cases Selection

We retrospectively reviewed the records of four patients with IVLBCL from December 2011 to July 2019 at Xiangya Hospital of Central South University. The clinical history, laboratory data, imaging abnormalities, treatment regimens, and outcomes were collected. The diagnosis of each case was confirmed with morphologic examination, immunohistochemical stains, or molecular studies. We also conducted a comprehensive literature review for the cases of IVLBCL published in the last 20 years (between 1999 and 2021) in PubMed (http://www.ncbi.nlm.nih.gov/pubmed/) using combinations of keywords in the title/abstract field, including “intravascular large b-cell lymphoma and IVLBCL”. The cases in the literature were carefully reviewed to extract necessary clinicopathologic data and combined with the cases repeatedly studied in different papers. A total of 311 cases of IVLBCL were retrieved from the literature and were included in our research (3–91).

### Specimen Processing and Immunohistochemical Staining

Computed tomography (CT)–guided percutaneous liver, lung, and adrenal glands biopsies were performed. All biopsied specimens of four cases were formalin-fixed and paraffin-embedded (FFPE) and subsequently sectioned at 4.0 μm for hematoxylin-eosin staining and immunohistochemical study. Appropriate negative and positive controls were performed with satisfactory staining.

### *In Situ* Hybridization

Digoxigenin-labeled probe (Dako) was used to detect Epstein–Barr virus–encoded small RNA. The *in situ* hybridization (ISH) assays were performed on a Ventana Benchmark XT platform with an iViewBlue detection kit according to the manufacture’s manual (Ventana Medical Systems Inc., Tucson, AZ, USA).

### Fluorescence *In Situ* Hybridization

Fluorescence ISH (FISH) studies were performed on two cases on FFPE tissue using C-MYC, BCL2, and BCL6 dual-colored break-apart rearrangement probes (Vysis Inc., IL, USA). Hybridization signals were assessed from 100 nuclei of tumor cells with an established cutoff of 15% for rearrangement of the gene locus. When the proportion of tumor cells with separation signals was less than 15%, the gene was interpreted as not rearranged. The FISH images were obtained using an Olympus BX51 fluorescence microscope and were captured on the Olympus Image DP2-BSW Software (Olympus Soft Imaging GmbH, Hamburg, DE).

## Results

### Clinical Characteristics

**Figure 1:**
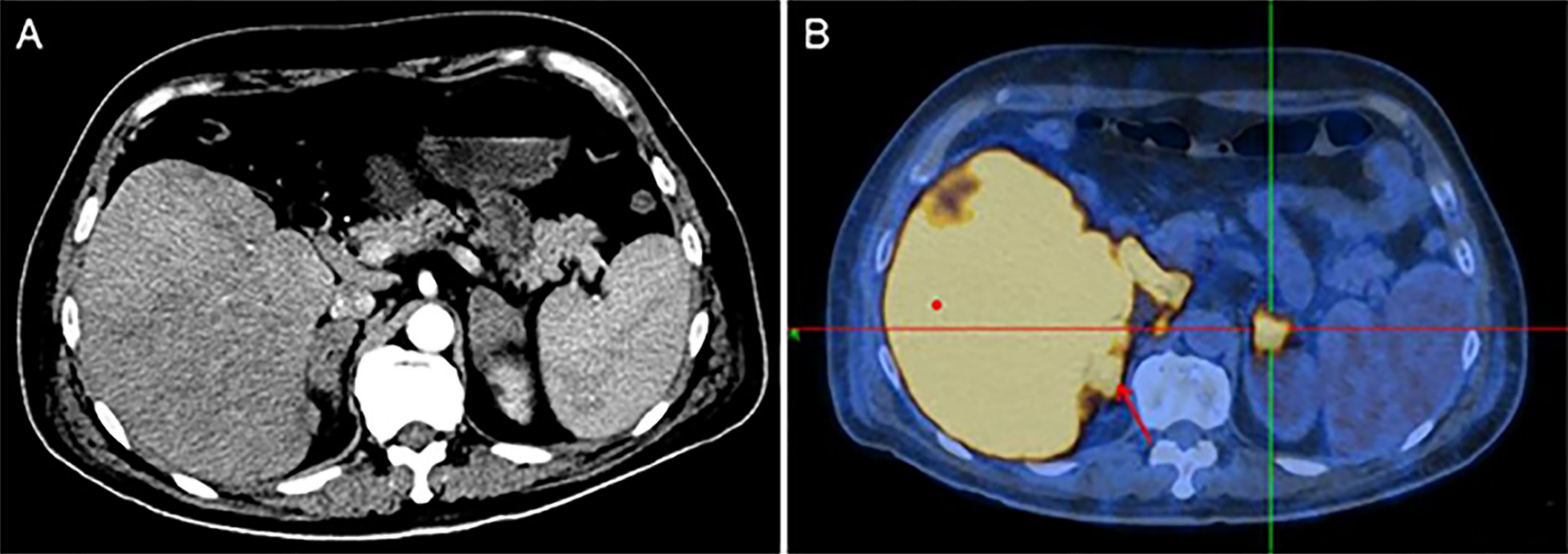
**(A)** Abdominal CT with contrast enhancement image showed diffuse enlargement of the liver, spleen, and bilateral adrenal glands. **(B)** PET-CT showed high uptake in liver and bilateral adrenal glands.

The major clinical features of the four cases of IVLBCL are presented in **Table 1**. Patients were middle-aged and elderly with an average age of 56 years at diagnosis ranging from 41 to 72 years and a male-to-female ratio of 1:1. The sites of initial diagnosis of IVLBCL were in the adrenal glands (two cases) and the liver and lung (one each case). All patients presented with B symptoms, including fever, night sweats, weight loss, and fatigue. Laboratory examination results revealed anemia (three of four), hypoalbuminemia (four of four), thrombocytopenia (one of four), and remarkably elevated levels of serum LDH, erythrocyte sedimentation rate (ESR), and C-reactive protein (CRP). Abdominal CT with contrast enhancement showed enlargement of bilateral adrenal glands (three of four) and hepatosplenomegaly (two of four). CT scanning of the chest revealed pleural effusion (three of four), and one case showed ground glass opacities in bilateral lung fields (84). Two patients presented enlarged retroperitoneal lymph nodes at CT scan, which were not biopsied. Fluorine-18 fluorodeoxyglucose (18-FDG) positron emission tomography–CT (PET-CT) study showed high uptake in the liver and bilateral adrenal glands (**Figure 1**). Bone marrow biopsies were performed in two patients, showing an active proliferation of granulocyte with obvious toxic reaction, but no evidence of lymphoma infiltration was detected. However, a bone marrow biopsy in one of the patients showed hemophagocytosis. In addition, the other two patients without bone marrow biopsy showed no hemophagocytic syndrome in clinical symptoms and serological results. All patients were diagnosed as IVLBCL by percutaneous organ biopsy. Only one patient received systemic chemotherapy consisting of rituximab, cyclophosphamide, doxorubicin, vincristine, and prednisone (R-CHOP) and had a complete remission at least initially; two patients received supportive care; and one patient refused any treatment. A total of two patients died of disease during the short period of follow-up.

**Table 1:** Clinical features of four cases of IVLBCL.

Clinical features	Case No.			
	1	2	3	4
Sex/age (years)	M/65	F/41	M/72	F/45
Primary location	Liver	Adrenal glands	Adrenal glands	Lung
BM	Negative	ND	ND	Negative
Symptoms	Fever, fatigue, and hypoxemia	Fever	Fatigue and weight loss	Fever, fatigue, night sweats, and hypoxemia
HB (g/L)	85.0	116.0	101.0	96.0
RBC count	2.6 × 10^12^/L	4.0 × 10^12^/L	3.6 × 10^12^/L	2.8 × 10^12^/L
WBC count	2.8 × 10^9^/L	7.1 × 10^9^/L	2.6 × 10^9^/L	7.3 × 10^9^/L
Platelets	81.0 × 10^9^/L	188.0 × 10^9^/L	133.0 × 10^9^/L	167.0 × 10^9^/L
LDH(U/L)	2330.0	453.0	872.0	1991.8
ESR(mm/h)	44.0	102.0	48.0	97.0
CRP(mg/L)	139.0	49.5	79.0	14
Albumin(g/L)	25.7	36.0	39.1	28.4
Ferritin (ng/ml)	2000.0	ND	860.0	ND
CT	Hepatomegaly, enlarged adrenal glands, and pleural effusion	Large adrenal glands	Hepatomegaly, enlarged adrenal glands, and pleural effusion	Bilateral ground-glass opacities of lung and pleural effusion
PET-CT	Liver and adrenal glands with FDG uptake	ND	ND	ND
Treatment	ST	ND	ST	R-CHOP
Follow-up	1 month	6 months	3 months	3 years
Outcome	Died	Alive	Died	LTF

M, male; F, female; BM, bone marrow involvement; HB, hemoglobin; LDH, lactate dehydrogenase; ESR, erythrocyte sedimentation rate; CRP, C-reactive protein; CT, computed tomography; PET, positron emission tomography; FDG, ^18^F-fluorodeoxyglucose; R-CHOP, rituximab, cyclophosphamide, doxorubicin, vincristine, and prednisone; ND, not done; ST, supportive therapy; LTF, lost to follow-up.

### Morphologic Features

**Figure 2:**
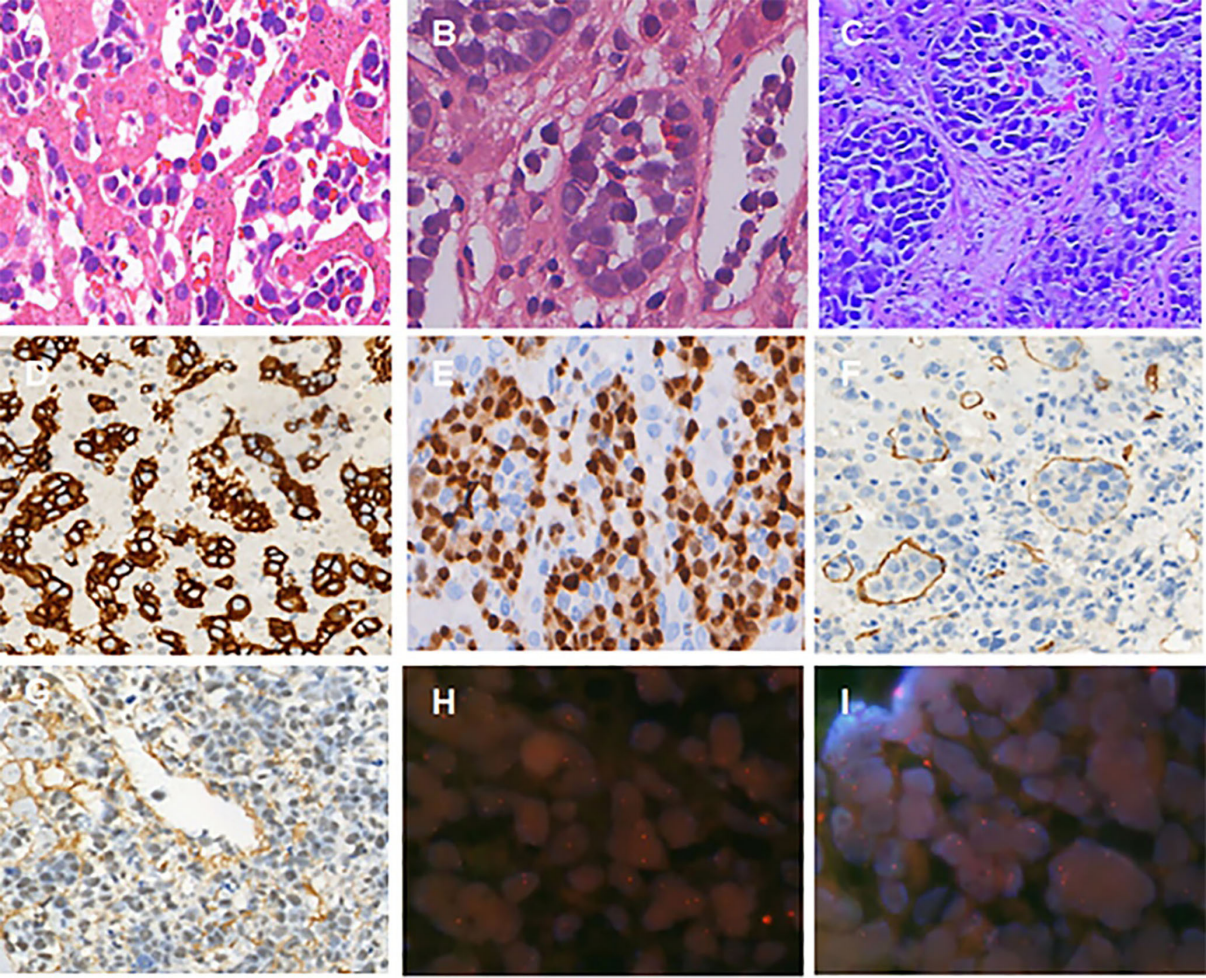
Histologic features of IVLBCL from the percutaneous organ biopsy specimens. **(A)** A liver biopsy demonstrated intermediate to large cells within sinusoids with irregular nuclei, dispersed chromatin, occasional nucleoli, and scant cytoplasm (case 1). **(B)** Cellulose thrombosis could be found (case 2). **(C)** Lymphoma cells were large, with scant cytoplasm, prominent nucleus, and frequent mitotic figures (case 3). **(D–G)** Immunophenotypic features of IVLBCL. The neoplastic cells were positive for CD20, MUM1, CD31, and EBER (original magnification D-G, ×200); FISH study showed non-rearrangement of *C-MYC*, *BCL2*, or *BCL6* gene on the IVLBCL cells. Non-rearranged gene is represented by orange/green fusion signal **(H, I)**.

All cases showed selective growth of large, atypical lymphocytes within the lumen of small blood vessels. Various degrees of cellulose thrombosis were observed in two cases. The neoplastic lymphocytes were large in size with one or more nucleoli, scant cytoplasm, and frequent mitotic figures (**Figure 2**).

### Immunohistochemistry and ISH

The results of immunohistochemistry and EBER-ISH are summarized in **Table 2**. Tumor cells were positive for CD20 (four of four), PAX5 (four of four), MUM1 (four of four), BCL6 (three of four), BCL2 (one of four), and CD5 (one of four), but negative for CD3 and CD10. The Ki-67 proliferative index ranged from 70% to 80%. ISH for EBER (Epstein–Barr virus-encoded small RNA) was positive in one patient (**Figure 2**).

**Table 2:** Immunohistochemistry, ISH, and FISH studies of the four cases of IVLBCL.

	Case No.			
	1	2	3	4
CD20	+	+	+	+
PAX5	+	+	+	+
MUM1	+	−	+	+
BCL2	−	+	−	−
BCL6	+	+	−	+
CD3	−	−	−	−
CD5	+	−	−	−
CD10	−	−	−	−
Ki-67	80	80	70	75
EBER ISH	+	−	ND	ND
*C-MYC* FISH	−	ND	ND	−
*BCL2* FISH	−	ND	ND	−
*BCL6* FISH	−	ND	ND	+

ND indicates not done.

### ISH Study

The C-MYC, BCL2, and BCL6 gene translocation were assessed using the dual-colored break-apart rearrangement probes in two cases (**Table 2**). In our study, rearrangement of BCL6 gene on the IVLBCL cells was detected in case 4, which had been reported previously (83). However, FISH study did not show any rearrangement of C-MYC, BCL2, and BCL6 genes in case 1 (**Figure 2**).

## Discussion

IVLBCL is a rare large B-cell lymphoma characterized by multifocal and selective growth of capillaries rather than arteries and veins. It primarily affects elderly individuals and patients usually without lymphadenopathy. Symptoms and signs are related to organs that block small blood vessels. The most commonly involved organs are the skin, central nervous system (CNS), and bone marrow (2). Tumor cell are positive for commonly used B-cell markers (CD20, CD79a, and PAX5), and aberrant expression of the T-cell marker CD5 is detected in a subset of cases.

There are some drawbacks in this study including the following: The number of cases is inadequate and clinical examination items were requested non-uniformly. Furthermore, the patient treatment information summarized from the literature is incomplete, and the follow-up duration is not enough. However, all the cases that we reported had typical clinicopathological and immunohistochemical features of IVLBCL. Notably, we noticed important findings in four cases. EBER-positive nuclear signals were detected by ISH in our case. Although Epstein-Barr virus (EBV)-positive DLBCL was listed as a distinct subtype of DLBCL in the revised World Health Organization classification (89). Cases reporting EBV-positivity in the liver are very rare. Few studies focusing on EBV expression in IVLBCL have been reported.

In the past, the majority of IVLBCL cases were diagnosed by autopsy. With the improvement of awareness of this entity, most patients were diagnosed by bone marrow biopsy and skin biopsy from positive skin lesions or random skin biopsy (RSB) (90). The liquid biopsy has also been reported as a promising method for diagnosing IVLBCL (85). In our study, no evidence of lymphoma infiltration was detected by bone marrow biopsy. However, we used CT-guided percutaneous organ biopsy as our diagnostic method of choice, including the adrenal gland biopsy (two cases) and the liver and lung biopsies (one each case). CT-guided percutaneous biopsy is a safe and accurate diagnostic procedure in IVLBCL.

Imaging study is helpful to the diagnosis of IVLBCL. A few cases reported PET-CT had been used for making initial diagnosis of IVLBCL by identifying appropriate target sites for biopsy, as these patients usually show high FDG-uptake in involved organs (85). In addition, the distribution and intensity of FDG uptake lesions on PET/CT can be used to predict outcomes and survival rates (86). In this study, two patients’ lesions were detected by PET-CT in the liver and adrenals. Moreover, CT showed ground-glass opacities of lung, hepatomegaly, and enlargement of adrenals. Some previous studies showed high frequency of neurological abnormalities on brain magnetic resonance imaging (MRI), and these abnormal findings appeared to relate to ischemic changes of small brain capillaries due to neoplastic lymphoma cells (27–35). However all patients in four new cases did not present neurological symptoms, and thus, brain MRI was not performed.

With standard chemotherapy of R-CHOP or autologous stem cell transplantation (SCT), about 60% of patients with IVLBCL was possible survived more than 5 years (90). In the present study, all patients were clinically suspected of and diagnosed with IVLBCL by two experts and received follow-up at our hospital. Importantly, a patient treated with R-CHOP has a survival time of at least 3 years.

## Conclusion

We described a comprehensive approach for the diagnosis and treatment of patients with IVLBCL. We also described peculiar clinical, laboratory, imaging, morphologic, and molecular features of this disease. We point out that this study provides valuable observations for recognizing the clinicopathologic features of IVLBCL and contributes to the early diagnosis of IVLBCL, which will increase the possibility of prompt treatments and ultimately lead to a better survival rate.

### Review of 311 Cases of IVLBCL

After an extensive search of the literature, we found 311 cases of IVLBCL. The detailed clinicopathologic data of the 311 cases from the literature are included in **Supplementary Tables** (**Supplemental Digital Content 1**, http://links.lww.com/PAS/A438).

IVLBCL mostly affected elderly patients with a male-to-female ratio of 1:1. The average age and median age were 65.2 and 67 years, respectively, ranging from 23 to 92 years. Diagnosis was established *in vivo* in 258 of 311 (83.0%) patients and postmortem in 51 patients (16.4%). Patients with IVLBCL were diagnosed *via* RSB (81 of 311, 26.0%), bone marrow biopsy (46 of 311, 14.8%), lung biopsy, and adrenal biopsy. IVLBCL lesions were found in almost all organs including the thyroid, liver, pancreas, prostate, and brain. Bone marrow lymphoma infiltration was detected in 92 patients (92 of 204, 45.1%). Characteristic features included persistent fever (190o of 199, 95.6%), hypoxemia (91 of 92, 99.0%), impaired consciousness (48 of 48, 100%), hypoalbuminemia (41 of 45, 91.1%). The clinical manifestations are mainly divided into two subtypes: one is the classic type and the other is the Asian variant. In Western countries, patients with IVLBCL usually show symptoms of CNS and skin involvement in 311 literature cases. In contrast, patients with IVLBCL in Asian countries usually present hemophagocytic syndrome, mainly including pancytopenia and hepatosplenomegaly. Among the laboratory abnormalities, anemia, and thrombocytopenia were the most frequently observed findings. Most patients exhibited extremely low levels of serum albumin, high levels of serum LDH, ESR, CRP, sIL2R, and ferritin. The increased level of serum LDH was present in almost 97% of cases, whereas anemia was present in two-thirds of cases.

Baseline imaging studies included whole body CT scan, brain MRI, and PET-CT in 180, 73, and 75 patients, respectively (**Table S2**). Prominent lymphadenopathy was not detected in the majority patients, but small groin or intra-peritoneal lymphadenopathy (<2 cm in diameter) was often observed. Splenomegaly and adrenal tumors were detected in 66 (66 of 180, 36.7%) and 11 (11 of 180, 6.1%) patients, respectively. Chest CT detected pleural effusion in 17 (17 of 180, 9.4%) and ground glass opacity of lung field in 35(35 of 180, 19.4%) patients. Brain MRI showed abnormal findings in 55 patients (55 of 73, 75.3%), with hyperintense lesions in the pons in three (3of 73, 4.1%), non-specific white matter lesions in eight (eight of 73, 11.0%), infarct-like lesions in four (four of 73, 5.5%), and meningeal enhancement in five (five of 73, 6.8%) patients. PET-CT revealed a high incidence of bone marrow (three of 75, 4%), bone (16 of 75, 21.3%), spleen (21 of 75, 28%), lymph node (two o 75, 2.7%), and adrenal gland (11 of 75, 14.7%) involvement.

The results of immunohistochemical analysis of markers for most patients remained consistent. In these statistics, most cases expressed CD20 (273 of 276, 99.0%), CD79a (112 of 114, 98.2%), and other whole B cell markers and expressed MUMI (85 of 100, 85%), BCL2 (62 of 76, 81.6%), CD5 (64 of 142, 45.1%), CD10 (16 of 135, 11.9%), and BCL6 (40 of 90, 44.4%). T cell marker CD3 (2 of 124, 1.6%) is negative. The expression of CD20 is negative, and the expression of CD3 is positive in a very small number of patients, which brings difficulties to the correct diagnosis. The proliferation activity of Ki-67 ranges from 20% to 100%. It has been reported in the literature that the proliferative activity of Ki-67 is an independent risk factor for the prognosis (survival time) of patients with IVL (88). The immunophenotype of most patients (68 of 83, 81.9%) is of non-germinal center origin (non-GCB). Studies have shown that the simultaneous expression of C-MYC and BCL2 may be a useful prognostic indicator. Compared with non-dual expressions, C-MYC/BCL2 dual expressions have a significantly higher mortality rate (88). Positive EBER expression accounts for only four (four of 52, 7.7%) patients.

Molecularly, chromosomal abnormalities were detected in bone marrow samples from the 34 patients in whom karyotype analysis was successful. A common pattern of abnormality could not be defined due to the heterogeneous and complex karyotype pattern of the disease and the small sample size (90). Noticeable, abnormality of chromosome 18 (four of 24, 16.7%) accounts for the largest proportion of chromosome abnormalities in patients with successful karyotype analysis in this study.

Therapeutic management of 220 patients is summarized in **Table S2**. A total of 220 patients were treated in hospital. Among them, 146 patients received targeted therapy based on the CHOP regimen, and most of them have received different doses of drug adjuvant therapy. Ten patients received at least one course of R-CHOP (median, six cycles), and then, methotrexate (MTX) was injected intrathecally during treatment for each course. In a few cases, hematopoietic SCT, surgery, and radiotherapy were selected as the treatment plan, and the treatment effect was also significantly improved. Because most of the cases did not mention the follow-up time and prognosis of patients after treatment, the overall survival rate of patients could not be calculated in this paper.

This review is the largest to date on clinical presentation forms, pathological features, therapeutic management, and outcome in patients with IVLBCL from the literature.

## Data Availability Statement

The original contributions presented in the study are included in the article/**Supplementary Material**. Further inquiries can be directed to the corresponding authors.

## Ethics Statement

Written informed consent was obtained from the minor(s)’ legal guardian/next of kin for the publication of any potentially identifiable images or data included in this article.

## Author Contributions

QL and YH designed the study, performed the statistical analysis, and wrote the manuscript. All authors contributed to the article and approved the submitted version.

## Funding

This work was partially supported by the National Natural Science Foundation of China (project no. 81602167), the Hunan Provincial Natural Science Foundation of China (project nos. 2017JJ3494 and 2021JJ31100), and the Science and Technology Program Foundation of Changsha City (project no. kq2004085).

## Conflict of Interest

The authors declare that the research was conducted in the absence of any commercial or financial relationships that could be construed as a potential conflict of interest.

## Publisher’s Note

All claims expressed in this article are solely those of the authors and do not necessarily represent those of their affiliated organizations, or those of the publisher, the editors and the reviewers. Any product that may be evaluated in this article, or claim that may be made by its manufacturer, is not guaranteed or endorsed by the publisher.
